# Improving kidney care for people with severe mental health difficulties: a thematic analysis of twenty-two healthcare providers’ perspectives

**DOI:** 10.3389/fpubh.2023.1225102

**Published:** 2023-06-28

**Authors:** Clodagh Cogley, Claire Carswell, Jessica Bramham, Kate Bramham, Aoife Smith, John Holian, Peter Conlon, Paul D’Alton

**Affiliations:** ^1^School of Psychology, University College Dublin, Dublin, Ireland; ^2^Department of Health Sciences, University of York, York, United Kingdom; ^3^School of Nursing and Midwifery, Queen’s University Belfast, Belfast, Northern Ireland, United Kingdom; ^4^King’s College London, London, United Kingdom; ^5^Irish Kidney Association, Dublin, Ireland; ^6^St Vincent’s University Hospital, Dublin, Ireland; ^7^Beaumont Hospital, Dublin, Ireland

**Keywords:** kidney disease, mental health, mental illness, health inequities, schizophrenia, dialysis, transplantation, kidney failure

## Abstract

**Introduction:**

People with severe mental health difficulties (SMHDs) and concurrent kidney disease have less access to quality kidney care and worse clinical outcomes. Our research investigates the barriers and facilitators to effective kidney care for people with SMHDs, and how care might be improved for this underserved population.

**Methods:**

We conducted semi-structured interviews with twenty-two physical (*n* = 14) and mental (*n* = 8) healthcare professionals with experience working with people with SMHDs and concurrent kidney disease. Interview data were analysed and interpreted using reflexive thematic analysis.

**Results:**

Four themes were generated from the data: 1. *“It’s about understanding their limitations and challenges, without limiting their rights”* describes how some people with SMHDs need additional support when accessing kidney care due to challenges with their mental state, motivation, cognitive difficulties, or mistrust of the healthcare system. 2. “*There are people falling through the cracks*” describes how the separation of physical and mental healthcare, combined with under-resourcing and understaffing, results in poorer outcomes for people with SMHDs. 3. “*Psychiatry is a black spot in our continuing medical education*” describes how many renal healthcare providers have limited confidence in their understanding of mental health and their ability to provide care for people with SMHDs. 4. “*When they present to a busy emergency department with a problem, the staff tend to go ‘…psych patient”*” describes how stigma towards people with SMHDs can negatively impact quality of care.

**Conclusion:**

Healthcare professionals accounts’ describe how people with SMHDs and kidney disease can have favourable outcomes if they have appropriate hospital, community and social supports. Findings indicate that effective management of kidney disease for people with SMHDs requires integrated physical and mental health care, which takes an individualised “whole person” approach to addressing the interaction between kidney disease and mental health.

## Introduction

People with severe mental health difficulties (SMHDs), including schizophrenia, psychosis, bipolar disorder and major depression, die an average of 15–20 years earlier than the general population (1). The majority of deaths in people with SMHDs are due to preventable physical conditions, such as diabetes, cardiovascular disease and kidney disease (2–4). There is growing evidence that unequal access to healthcare plays a critical role in this disparity. Numerous barriers to accessing healthcare for people with SMHDs have been identified, including cognitive functioning, lack of social support, difficulty communicating healthcare needs, and fear or suspiciousness towards healthcare providers (5). Pervasive stigma regarding SMHDs and the separation of mental healthcare from other medical services also contribute to this disparity (6).

Chronic Kidney Disease occurs when damage to the kidneys impairs their ability to filter blood. This results in the accumulation of excess waste and fluid in the body, leading to other health problems such as cardiovascular disease and stroke (7). Kidney failure, also known as end-stage kidney disease, occurs when the kidneys can no longer adequately filter the blood, and kidney transplant or dialysis are needed for survival. Kidney failure carries a substantial and unique burden in terms of self-management and adherence. People on haemodialysis must undergo treatment several times a week for 4 hours at a time, and restrict fluid intake to 500mls per day (8). Failure to attend even one session may be life-threatening (9). This, combined with treatment side-effects, can significantly impact quality of life and ability to work or engage in other activities (10). Additionally, people on haemodialysis must adhere to a strict diet to prevent health complications. This diet can be extremely challenging, and restricts intake of foods high in sodium, potassium, phosphorous, protein and fluid (11). Kidney transplant is the optimal treatment for kidney failure as it increases survival and significantly improves quality of life (12). However, kidney transplant recipients must also be able to adhere to life-long medication regimens, lifestyle changes and medical appointments to sustain graft function (13).

The prevalence of kidney disease is higher in people with SMHDs (14). This is due to a range of factors, including the use of psychotropic medications such as lithium, as well as higher rates of smoking, type 2 diabetes and cardiovascular disease (14–20). However, available data regarding the rates of SMHDs in people with kidney disease is limited. In the United States, rates of psychiatric hospitalisations are 1.5 to 3 times higher in people with kidney failure compared to those with other chronic illnesses (21), and 27% of Medicaid-enrolled adults with kidney failure have been hospitalised with a psychiatric diagnosis (22). Recent data from Ireland suggests the prevalence rates of bipolar disorder, psychosis and schizophrenia diagnoses in people with kidney disease are 2.2, 1 and 1%, respectively (23). However, as this study relied on self-report surveys, it likely underestimates the true prevalence of SMHDs in people with kidney disease.

After developing kidney disease, people with SMHDs also have poorer clinical outcomes and limited access to kidney care. People with kidney failure and co-occurring SMHDs have higher risk of mortality and hospitalisations, particularly through the emergency department (22, 24, 25). There is also evidence that people with SMHDs are more likely to die before reaching the later stages of kidney disease (26). Furthermore, people with SMHDs are less likely to receive a kidney transplant (19, 27). This is despite evidence that, following a careful selection process, people with SMHDs have comparable kidney transplant outcomes to those without SMHDs (28–30). People with kidney failure and co-occurring SMHDs are also less likely to receive appointments with renal clinicians (nephrologists) or erythropoietin prescriptions (26). Research in other long-term conditions illustrates that people with SMHDs often have difficulty adhering to medications, diet, and fluid restrictions (31). Given the significant burden of adherence in kidney disease (32), people with SMHDs likely experience specific challenges in receipt of kidney care.

Despite the higher risk for kidney disease in people with SMHDs and the observed poorer outcomes for this population, research investigating kidney care for people with SMHDs is limited. To our knowledge, the only published research in this field is a qualitative study exploring the experiences of renal nurses providing acute haemodialysis to people with SMHDs, which did not focus specifically on barriers or facilitators to care (33). Nurses in this study described working with people with SMHDs as “challenging,” and identified staff shortages and lack of staff education regarding mental health as barriers to care. Participants described empathy and effective communication skills as facilitators to care, and expressed a need for more support from mental health teams. As healthcare providers work within healthcare systems and provide care to many patients over significant periods of time, they likely have insights into a range of barriers and facilitators to kidney care for people with SMHDs, as well as practical suggestions regarding how care for this population might be improved. As care for people with SMHDs is complex and multidimensional, research exploring the perspectives of a range of healthcare professionals from hospital and community settings is needed to inform the provision of kidney care for this population.

To better understand the barriers and facilitators to effective kidney care for people with SMHDs, we interviewed healthcare and mental healthcare professionals with experience working with people with SMHDs and concurrent kidney disease. It is hoped that our findings will contribute to improvements in kidney care for people with SMHDs and ultimately improve clinical outcomes for this underserved population.

We aimed to address two research questions:

- What are the barriers and facilitators to effective kidney care for people with SMHDs, from the perspective of healthcare and mental healthcare providers?
- How might kidney care for people with SMHDs be improved?

## Methods

This study was conducted as part of a larger research project investigating kidney care for people with SMHDs. Design and implementation of the wider research project were influenced by a PPI advisory group of three individuals with kidney disease and concurrent mental health difficulties. We adopted a qualitative approach using semi-structured interviews. This ensured that participants were asked the same broad, open-ended questions related to barriers and facilitators to effective kidney care for people with SMHDs, while allowing flexibility to discuss issues other than those predetermined by the researchers. The interview schedule was developed based on existing literature regarding inequities in access to healthcare for people with SMHDs (5) and access to kidney care for people from other minority backgrounds (34). The schedule was then informed by guidance from the PPI advisory panel and a Consultant Nephrologist (KB) (See Supplementary Appendix A for interview schedule).

### Background to pathway of care for kidney disease in Ireland

All people living in Ireland are entitled to receive healthcare though the public healthcare system, which is managed by the Health Services Executive (HSE). Both public and private healthcare services are available, and a minority of people with kidney disease have private consultations with nephrologists that are reimbursed by their private health insurance. However, the bulk of the costs are borne by the State, particularly for dialysis treatment (35). All adults with kidney failure in Ireland are under the clinical governance of 1 of 11 HSE renal units. In Ireland, people with kidney disease have access to a highly trained workforce of multi-disciplinary clinicians, and to the most modern equipment and medications (36).

Many people with kidney disease attend nephrology clinics for years before developing kidney failure. During this time, nephrologists monitor kidney function and slow the progression of kidney disease. In some cases, individuals first present to nephrology services with advanced kidney disease and require dialysis immediately. These late referrals or “crash landers” typically have worse health outcomes (37). Of the 5,148 people with kidney failure in Ireland, 52% have a functioning kidney transplant, the optimal treatment for kidney failure (35). However, not all people are eligible for transplant due to various reasons such as medical conditions or lack of suitable donors. Those who are not eligible for transplant must undergo dialysis to stay healthy. In Ireland, 41% of people with kidney failure are treated by centre-based haemodialysis, and 6% by home-based modalities (peritoneal dialysis or home haemodialysis) (35). Most people waiting for a kidney transplant will be on dialysis, although some can undergo a pre-emptive transplant before requiring dialysis. A retrospective study indicated that 45% of people transplanted in Ireland between 1996 and 2000 had their kidney transplant last for 15 years or more (38). After the graft fails, people typically return to dialysis. Some individuals are subsequently able to receive additional kidney transplants after graft failure.

### System of care for SMHDs in Ireland

As a result of recent changes in mental health policy, almost all of the larger psychiatric hospitals in Ireland have been closed and replaced with smaller acute inpatient units and community-based mental health services (39, 40). However, there is frequent criticism that community mental health teams in Ireland are not developed or resourced enough to provide adequate community support, and have significant regional variation and inadequate multidisciplinary input (41, 42). There is also still a significant number of individuals with SMHDs who are repeatedly admitted to acute inpatient units and spend significant periods of time there (43). In response, specialist rehabilitation units were commissioned in 2018, to provide specialist inpatient rehabilitation and recovery services to people with SMHDs with ongoing complex needs (40). However, there is an absence of intensive care high support hostels, crisis houses, and specialist rehabilitation units in each regional area (43).

### Participants

Twenty two professionals from physical healthcare (*n* = 14) and mental health (*n* = 8) were recruited through three Dublin-based hospitals and the Irish Kidney Association (see Table 1 for participant demographics). The study was advertised in three Dublin-based hospitals and through the Irish Kidney Association, inviting healthcare professionals with experience working with people with kidney disease and co-occurring SMHDs to participate. Snowball sampling was also used, whereby participants referred other potential participants who had experience working with this population. The inclusion criteria were that the participant must be a healthcare or mental healthcare provider who has experience working with people with kidney disease and co-occurring SMHDs; be fluent in English; and able to provide informed consent.

**Table 1:** Summary of participant demographics.

Age	Mean: 44.66 years; Range: 28–68 years; SD: 10.46
Gender	16 female; 8 male
Role	4 Nurses in Haemodialysis
	3 Consultants of Liaison Psychiatry
	3 Clinical Nurse Specialists in Nephrology
	2 Consultants of Nephrology
	2 Registrars in Nephrology
	2 Dieticians in Nephrology
	2 Counselling Psychologists in Nephrology
	1 Consultant of Immunology
	1 Liaison Psychiatric Nurse
	1 Community Mental Health Nurse
	1 Clinical Psychologist in Nephrology
	1 Physiotherapist in Nephrology
Time spent in role	Mean: 8.3 years; Range: 6 months – 38 years

### Procedure

This study was approved by the University College Dublin Research Ethics Committee (HS-21-19- Cogley-Dalton). Participants completed a semi-structured interview with the first author (CC), in a quiet room or *via* a teleconferencing platform. The interviewer did not know the participants and there was no monetary compensation for participation. Interviews took place between August 2021 and May 2022, and lasted 35–120 min (*M* = 53 min). Interviews were digitally recorded and transcribed verbatim.

### Analysis

**Figure 1:**
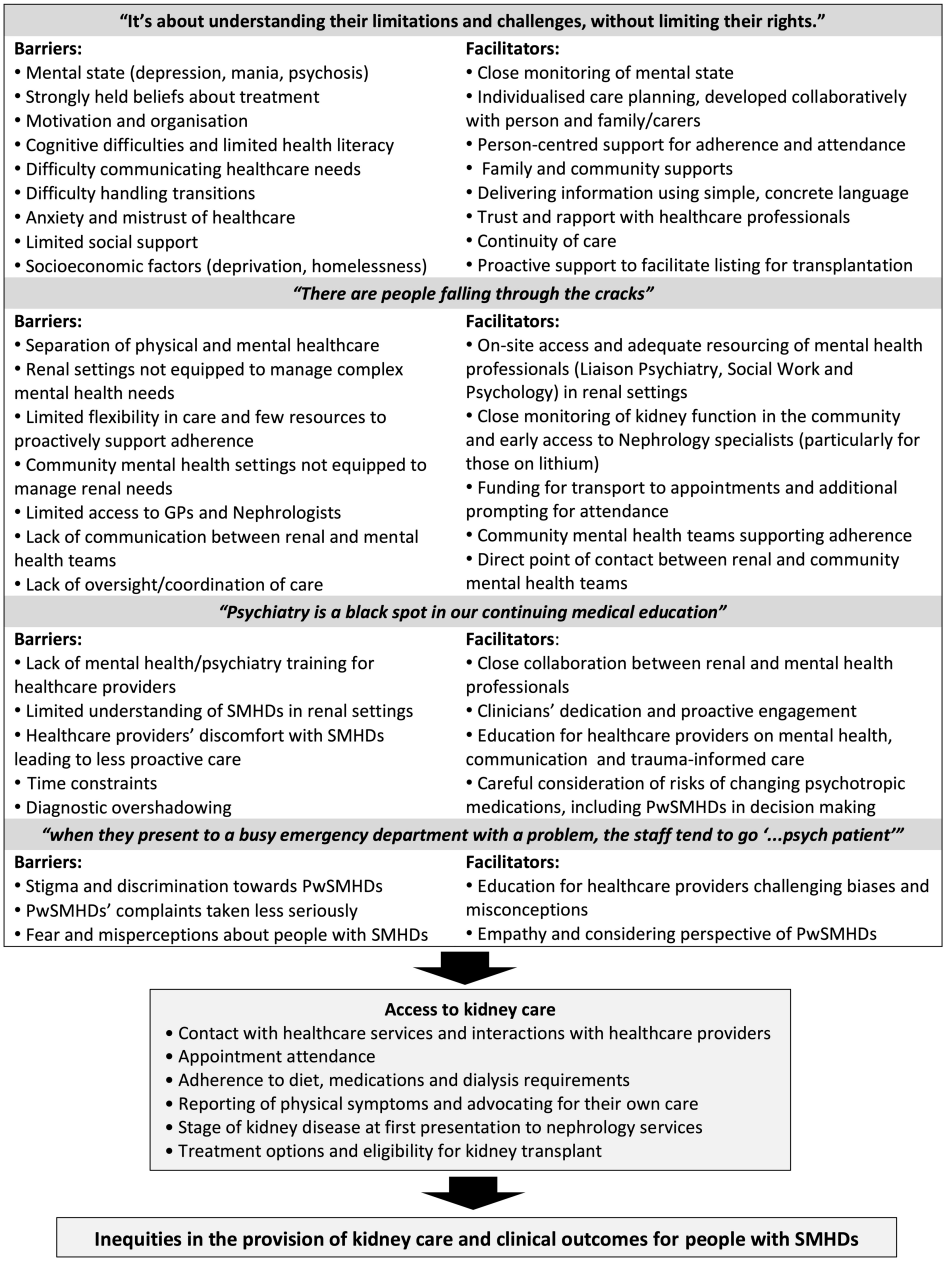
Summary of barriers and facilitators to effective kidney care for people with severe mental health difficulties (pwSMHDs).

Interviews were analysed using thematic using reflexive thematic analysis, using an adapted version of the procedures described by Braun and Clarke (44–46) (See Table 2 for a detailed description of procedures). Thematic analysis was chosen for its ability to analyze and capture the diverse experiences of participants with different roles (47). Analysis was conducted within a critical realist framework (48), interpreting participants’ accounts as “real” without rendering them independent of the cultural, disciplinary or political context in which they occurred. The use of thematic analysis was descriptive and sought to “stay close” to participants’ descriptions. We adopted a predominantly inductive approach, by open-coding data and emphasising data-based meaning. A degree of deductive analysis was employed to ensure the themes were relevant to the research questions (see Figure 1). Braun and Clarke describe their approach to thematic analysis as “reflexive” as it emphasises the active role of the researcher in the interpretation and generation of themes (49). Their gauges of quality include researcher reflexivity, systematic and rigorous coding, and theoretical knowingness (46). To monitor the impact of assumptions and biases throughout data collection and analysis, CC kept a reflexive journal and discussed her reactions and assumptions with two academic supervisors who work in Clinical Psychology (PD and JB). CC is a PhD researcher with a background working in health psychology. CCa is a post-doctoral researcher with background training in mental health nursing and with multiple years of experience working with people with SMHDs and people with kidney disease.

**Table 2:** Process of thematic analysis.

Phase	Process and author involvement
Phase 1: Data familiarisation	CC conducted interviews, keeping notes of insights and impressions. Interviews were transcribed verbatim. CC removed potentially identifying information and assigned pseudonyms to participants. CC read the data multiple times to facilitate deep familiarisation, and discussed impressions with JB and PD
Phase 2: Coding	CC and CCa independently and systematically conducted open coding and subsequently discussed findings, to foster reflexivity, enhance understanding, and improve “trustworthiness” of the analysis. Coding was an iterative process, staying close to participants’ accounts and primarily focusing on semantic meaning. Codes were stored and organized using NVivo 12 software
Phase 3: Generating initial themes	CC generated initial themes relating to barriers and facilitators to kidney care for people with SMHDs, by clustering together related or similar codes based on patterns of shared meaning
Phase 4: Reviewing and refining themes	CC, CCa, PD and JB reviewed initial themes. Through discussion, themes were discarded or reworked until they were seen as providing a “good fit” with the data. CC reviewed the themes against the coded data and the entire dataset
Phase 5: Defining and naming themes	CC refined and developed the “story” of each theme and subtheme, finalising theme names and writing the results section in full. These were reviewed by other authors
Phase 6: Producing the report	CC selected illustrative extracts which other authors reviewed and agreed upon. CC wrote the final paper, with input from all other authors

## Results

Four overarching themes were generated from the data, relating to barriers and facilitators to kidney care for people with SMHDs, and how kidney care might be improved for this population. A summary of the identified barriers and facilitators to care are illustrated in Figure 1.

## “It’s about understanding their limitations and challenges, without limiting their rights”

Participants reported that many people with SMHDs require additional support due to issues with their mental state, motivation and organisation, cognitive difficulties, or mistrust of the healthcare system. As the difficulties experienced by each person with a SMHD varies widely, participants emphasised the importance of understanding the specific needs of each individual, so they can be given appropriate support to access kidney care.

### Fluctuating mental state impacts engagement in kidney care

Depression was described by renal healthcare providers as the most common mental health-related barrier to care, impacting peoples’ ability to engage and adhere to treatment recommendations: “It becomes clear the reason why they are not able to do things at home is because they are so depressed they do not have any motivation to cook food.” (Laura, dietician). Haemodialysis nurses reported that a number of their patients had died by suicide, and expressed concern about the high risk of death for people who miss dialysis or do not adhere to diet guidelines: “If they want to die, they know how to die. Just eat 10 bananas” (Lucas, Haemodialysis Nurse).

Participants also described how individuals experiencing mania or psychosis can struggle to sit through a full dialysis session. Nurses discussed the difficulties they face when administering haemodialysis to people with SMHDs who are restless or agitated: “That needle can dislodge and hurt the patient, so much blood goes everywhere. And the patient is at risk of dying.” (Mia, Haemodialysis Nurse). In addition, some hold strongly held beliefs about their treatment or specific healthcare providers that can impact adherence:

*People with bipolar, if they're a bit high, they say “There's nothing wrong with me. I don't need that.”* – Liam, Liaison Psychiatrist

*We have people who think dialysis will end up killing them, or if we give them a certain injection it will give them heart failure. And then they end up just refusing to show up to dialysis* – Elijah, Haemodialysis nurse

In addition to posing risks to their health, fluctuating mental state was described as a barrier to transplant for some people with SMHDs, as poor medication adherence and appointment attendance post-transplant is associated with high risk of kidney rejection. One nephrology consultant described the difficulty of weighing the potential benefits and risks of putting a people with SMHDs forward for transplant when there are concerns regarding adherence:

*The conflict of my heart is am I, as a responsible senior clinician, going to refer this man to a transplant program? (…) Is he going to take his tablets every day? Is he going to end up rejecting that kidney and be worse off than he was before?* – David, Nephrology Consultant

Participants also discussed the risk of steroid-induced psychosis post-transplant, and emphasised the need for close monitoring during transition periods, to enable immediate intervention if necessary. Participants described the need to have an individualised advance care plan for people with fluctuating mental states, including specific guidance on how treating clinicians should respond if the person becomes acutely unwell:

*In a cardiac arrest situation, everybody knows what to do. You go in. You call a crash trolley. That's our job. What about if somebody had a severe change in their mental health? What do you do? Should there not be a similar protocol?* – Charlotte, Physiotherapist

They recommended that care plans be developed collaboratively by members of the renal MDT, mental health team, the person with the SMHD, and involve family and carers as appropriate. Participants described multiple instances whereby advance planning of care and supports were used to successfully facilitate people with SMHDs receiving a kidney transplant.

### Difficulties with motivation and organisation impact adherence and attendance

Mental healthcare providers described how low motivation can often be a feature of SMHD, which can result in some people with SMHDs being less proactive in their own care. Thus, they may require more proactive support to attend appointments:

*I think medics often think, "Well, if they don't turn up, that's their choice." And that's fine for some people. But when they have a lack of motivation as a part of their illness, that's when you need more prompting and more proactive engagement*. – Liam, Liaison Psychiatrist

Participants reported that adhering to the restrictive food and fluid guidelines can be particularly challenging for people with SMHDs on haemodialysis, and that external supports are sometimes necessary to sustain their adherence:

*The diet can be very bland and very difficult. And even the rigor that requires double boiling of vegetables and stuff like that. That’s hard with the best will in the world, not to mind if you’re struggling with motivation and organizational skills. (…) And for some, drinking large amounts of fluids is a component of their psychiatric disorder.* – Noreen, Liaison Psychiatrist

Conversely, participants described how other people with SMHDs can have high levels of health-related anxiety: “One would be very focused on numbers and be really worried if they heard that they had high potassium.” (Samuel, Registrar in Nephrology). As a result, some people with SMHDs may follow treatment guidelines too rigidly, which can also lead to health complications:

*If you advise on something they might take that to the letter of the law, and sometimes that can be a good thing, but in the overall it might be driving anxiety and other issues. Some people might be overly compliant or overly restricted in their diet, and then that could turn into an issue as well.* – Laura, dietician

Participants also described how lack of organisation and motivation can result in under-reporting of physical symptoms in people with SMHDs. Alogia, a symptom of some SMHDs whereby a person has poverty of speech, can also contribute. As a result, people with SMHDs are more likely to seek medical attention when they are in a “physical health crisis” (Mia, Mental Health Nurse), which can lead to later diagnosis of kidney disease and poorer outcomes:

*You get the referral from the GP or the mental health services, but they have presented at a later stage and the kidney disease is already underway. So you're kind of catching up with what damage has been done.* – Evelyn, Advanced Nursing Practitioner in Nephrology

Mental healthcare participants described how the motivation of people with SMHDs to seek healthcare is also impacted by socio-economic factors, including limited health education and higher rates of poverty, homelessness and addiction in this population: “To be honest, attending some appointment is not going to be their priority when there is so much else going on in their lives, so many disadvantages.” (Elizabeth, Community Mental Health Nurse).

Because of difficulties with organisation and motivation, mental healthcare professionals described the need for external supports to help people with SMHDs manage their physical and mental health, navigate the healthcare system and attend appointments. Participants reported that support from family members is extremely helpful, but that many people with SMHDs have limited social supports to advocate for their care. Indeed, participants reported that the highest risk group of people with SMHDs are not those with the most significant impairments, but rather those who are not in fully supported accommodation and have limited family support:

*Adherence is more of an issue for the patients in that intermediate group who are more vulnerable, because they aren't well enough to be able to have routine social supports of family or partners, but aren't ill enough to be requiring full supports to help with medication changes post-transplant.* – Noreen, Liaison Psychiatrist

### Cognitive difficulties impact communication needs and treatment options

Mental healthcare providers described how some people with SMHDs have cognitive difficulties, including with information processing, disorganised thinking, literal thinking, memory, and attention. This impacts the ability of some people with SMHDs to understand kidney disease, adhere to treatment requirements, communicate with healthcare providers and navigate the healthcare system. As a result, treatments requiring higher levels of education and understanding, such as peritoneal dialysis or home haemodialysis, are less likely to be offered to people with SMHDs.

Participants highlighted the need to give people with SMHDs more time to communicate and take in information. However, resource restraints limit the time they can spend with people with SMHDs: “If I’m spending a lot of time with that one person, I cannot spend that time with somebody else, and that is challenging” (Charlotte, physiotherapist). Participants recommended individualizing the delivery of information, using simple language, direct and concrete communication styles, checking the persons’ understanding, and repeating information. Providing individualised information in a variety of formats, including verbal, written and video, was also recommended.

*I find it very helpful to write down information for people, writing down the date and management plan and individualizing it. So that patients can look at it afterwards and remember, "Oh, that was the plan because…"* – Noreen, Liaison Psychiatrist

Where possible, sharing information with carers and family members was also recommended, so they can act as an additional source of information to the person with the SMHD, and support their adherence to treatment recommendations at home. As the needs of each person with SMHDs are different, participants described how families often inform care plans and help healthcare providers know what to expect when providing care to people with SMHDs.

### Anxiety and mistrust impact their interactions with healthcare

Participants reported that many people with SMHDs have high levels of anxiety and mistrust, particularly when interacting with healthcare. The anxiety was attributed to previous negative experiences with medical establishments, such as being involuntarily detained, as well as being a feature of their SMHD. Mental health providers described how fear and anxiety often led to people with SMHDs avoiding healthcare appointments:

*It takes a lot of effort to get the person to go to the doctor. And if they don't get a positive response from the receptionist when they go in, they turn and walk out of the place.* – James, Liaison Psychiatric Nurse

Participants reported that people with SMHDs’ mistrust of healthcare providers led to not sharing important information regarding their health. High levels of anxiety also make it more difficult for people with SMHDs to listen to healthcare providers or engage in care: “when she’s not well, her anxiety levels kind of are just on the Richter scale. And she’s not really listening, she’s not able to.” (Evelyn, Clinical Nurse Specialist). Participants highlighted the importance of listening to each person’s concerns, showing empathy, and working to build a trusting relationship with them: “It’s about empathy, really listening to the person and trying to understand their distress. Even if it might not make much sense to us.” (James, Liaison Psychiatric Nurse). Participants also highlighted the value of trauma-informed care:

*We need to be trauma informed because a lot of people with psychosis do have a significant trauma history. It doesn't mean you are expected to delve into that, but, to be aware that this could be a significant factor.* – Jack, Community Mental Health Nurse

Having a predictable routine was described as helpful for many people with SMHDs. Participants reported that after adapting to dialysis, the attendance of people with SMHDs is often better than those without SMHDs. However, periods of transition, including the initiation of dialysis and transplant, are more challenging for people with SMHDs: “some of them have a low tolerance for change.” (Olivia, Nephrology Consultant). Participants stressed the value of continuity of care and the long-term relationships renal healthcare providers often have with their patients, as clinicians come to learn the specific needs of each people with SMHDs: “It can help because we can say ‘Look, that’s actually normal for them, and this is a good sign that they are reacting that way.’” (Charlotte, Physiotherapist). Similarly, matching patients to their preferred nurses and doctors can reduce anxiety and help care go more smoothly. Participants praised the dedication that renal teams show to their patients, and described how this is essential to ensuring people with SMHDs stay engaged in kidney care:

*I think renal teams try and do as much as they can to have people well enough for dialysis. There was one case in particular where he was quite psychotic, and very disengaged, and they really put a huge amount of work into seeing him, and engaging him. And even when he wouldn't engage with us, the renal team stuck with it, kept with him.* – Emma, Liaison Psychiatrist

Participants also discussed how rates of SMHDs are higher in people who are from minority backgrounds, including those who are not Irish, White, or who do not speak English as a first language. Participants described how people with SMHDs from minority backgrounds face additional barriers to kidney care, and may have additional mistrust of healthcare professionals, due to previous negative experiences due to lack of cultural awareness or racism: “Because of that bad treatment, there can be higher levels of fear and avoidance.” (Isabelle, Clinical Psychologist). To improve access to kidney care for this population, participants highlighted the need for healthcare providers to be responsive to cultural needs, as well as differing health beliefs and practices.

## “There are people falling through the cracks”

Participants stressed the interaction between physical and mental health, and described how the separation of physical and mental healthcare results in poorer outcomes for people with SMHDs.

Participants reported that people with SMHDs often have more complex physical health problems due to the use of psychotropic medications; health behaviours such as diet, smoking and drug addiction; as well as socioeconomic factors, such as higher rates of poverty and homelessness. Participants described how these health complications can impact treatment options and eligibility for a kidney transplant. Participants described how integrated physical and mental healthcare is necessary for effective care for people with SMHDs: “With better treatment of a person’s mental illness, the physical illness became much easier to manage. So you cannot separate them out and box them.” (Grace, Consultant Immunologist). However, participants reported that physical health settings are often not equipped to manage the needs of people with SMHDs:

*The system expects a certain type of person who is able to engage fully, is organized enough, has their transport and is able to get here with no issues. And not for vulnerable groups, people with mental health difficulties, the homeless, with not enough support.* – Grace, Consultant Immunologist

For example, participants described how renal departments are often not adequately resourced with liaison psychiatry, social work, psychology or clinical nurse specialists trained in mental health: “There is no dedicated psychiatry for the renal outpatient service, so unless someone is actually suicidal, they will not be coming down.” (Samuel, Nephrology Registrar). Interdisciplinary working between renal and mental health professionals were described as improving care for people with SMHDs: “We have a good working relationship with the medical Consultants, and when they ring me, I know that there’s something up, and we need to respond quickly.” (Emma, Liaison Psychiatrist). Similarly, participants highlighted the benefits of having on-site access to mental health staff:

*There was a man who they couldn't manage in a previous dialysis centre because of his behaviour. So he got transferred to our dialysis unit because of our ability to have enhanced interventions. So when he came initially he was having two staff sitting beside him throughout his dialysis, and now he comes in himself and has good working relationships within the context of a very complex psychiatric presentation. (…) And that's the process of liaison psychiatry, is that access to onsite psychiatry rather than calling somebody in from elsewhere who doesn't know the system, doesn't know the patients, doesn't know the staff, and isn't available to come in at the times when the patient is there on dialysis* – Noreen, Liaison Psychiatrist

Participants described how, because of time and resource constraints, there is often little flexibility in terms of how care is provided in Irish hospitals:

*We need to think, what is the target population we're looking at? How can we design it around that person and their needs? Because most healthcare systems are built around the professionals’ needs, not the patients’.* – Jack, Community Mental Health Nurse

For example, participants described how appointments are typically at set slots with little allowance for additional time. Similarly, while dialysis nurses often spend significant periods of time contacting patients, rescheduling appointments and organising transport to facilitate attendance, most pre-clearance clinics have limited resources to do this. This can result in people with SMHDs “falling through the cracks” (Elizabeth, Community Mental Health Nurse) and losing contact with healthcare services. Participants also described how the separation and specialisation of healthcare departments can result in a lack of oversight regarding the “bigger picture of care” (James, Liaison Psychiatric Nurse) of people with SMHDs:

*It's about the holistic care. And I think that's where healthcare in Ireland is really poor. Everyone does their own bit. And sometimes assumptions are made that another service is doing it, when they’re not.* – Evelyn, Advanced Nursing Practitioner in Nephrology

Similarly, mental health practitioners described how mental health settings are often inadequately resourced to support the complex health needs of people with SMHDs, carry out regular health screenings, or promote prevention. They reported that limited access to general practitioners (GPs) in the community often negatively impacts people with SMHDs’ access to physical healthcare, medical benefits, and referrals to mental health teams. Mental health participants were very aware of the impact of lithium on the kidneys, and reported they regularly monitor the bloods of people taking lithium-based medications. However, they described how access to Nephrology specialists is often limited to when the person’s kidney damage has already progressed. In the absence of specialist advice, mental health practitioners are often unsure of how to prevent further kidney damage: “You’re looking at their kidney function reducing and you think, oh god, what am I supposed to do now?” (Liam, Liaison Psychiatrist).

Participants described how close interdisciplinary working and communication between renal and mental healthcare professionals is needed to optimize care for people with SMHDs:

*We can’t just separate out and treat the mental health part, just like we can’t just treat the kidneys and not worry about the rest of the body. We need to treat the whole person, so the more integrated the different services can be the better things will be for the patient.* – James, Liaison Psychiatric Nurse

However, communication between community mental health and renal teams can be limited. Mental health participants described the difficulties of having separate physical and mental healthcare records, as well as not having a named person on the renal team to contact about concerns. Participants reported that liaison psychiatry can often act as a bridge between the physical and mental healthcare teams.

Under-resourcing of community mental health teams also impacts their ability to coordinate the physical and mental healthcare of people with SMHDs. For example, community mental health nurses described how they sometimes help people with SMHDs attend healthcare appointments by prompting or accompanying them. However, this does not occur routinely as it is not specified in their job descriptions, and the resourcing of local community mental health teams varies widely. Community mental health workers reported that they sometimes use their own taxi voucher books to ensure people with SMHDs can attend appointments: “Which I think is a little unfair because that means our budgets are being interfered with, but also we are managing complex conditions that are definitely outside our scope of practice.” (Jack, Community Mental Health Nurse).

## “Psychiatry is a black spot” in our continuing medical education

Most renal healthcare providers reported that they have a limited understanding of SMHDs, and had received little to no education regarding SMHDs since medical school: “Psychiatry is a black spot in our continuing medical education. My psychiatry education was probably highest in medical school.” (David, Consultant Nephrologist). Renal healthcare providers described having less training in psychiatry than in other specialities, and some admitted they sought it out less. Participants described how most healthcare providers are less comfortable working with mental health conditions that cannot be explained in “concrete and biological terms” (Samuel, Registrar in Nephrology), without a clearly defined intervention:

*For example, post-myocardial infarction, a person's mortality and morbidity is better predicted by their level of depression than by their left ventricular ejection fraction, but we like to measure left ventricular ejection fraction. We don't follow the evidence, we follow the technology and we steer away from what feels more vague and fuzzy. Even though with our increasing knowledge around the brain and neuroscience, now we have really solid evidence-based pathways to support ways forward*. – Noreen, Liaison Psychiatrist

Most renal healthcare providers reported that they do not fully understand the meaning of different SMHD diagnoses, or how they might impact the delivery of care for people with SMHDs. Because of this lack of understanding, combined with inadequate access to mental healthcare professionals and limited time to spend with patients, some renal healthcare providers described taking a “do not ask questions approach” (Samuel, Nephrology Registrar) to mental health. Participants reported that healthcare providers often felt anxious when interacting with people with SMHDs, due to lack of experience and education: “I’ve seen it a lot in acute hospitals, when they deal with people with mental disorders there’s often a lot of apprehension and discomfort, which is really unfortunate in my opinion.” (James, Liaison Psychiatric Nurse). Indeed, many renal healthcare providers who worked in a hospital with limited access to mental health professionals described feeling “helpless” (Lucy, Haemodialysis Nurse) when discussing the mental health needs of their patients:

*I think when it comes to mental health stuff though we sort of hold our hands up. It's almost like dentistry, we are never going near the mouth, that's just been cordoned off. We respond in a similar way to psychiatric stuff.* – David, Consultant Nephrologist

Mental healthcare professionals described how an important part of their role on the MDT is upskilling and increasing the confidence of physical healthcare providers when responding to the needs of people with SMHDs:

*It is about normalizing psychiatric brain disorders as a routine part of medical care. (…) It allows an increased proactivity, and an increased sense of confidence in the healthcare professionals, in terms of engaging actively in the patient's care* – Liam, Liaison Psychiatrist

Participants reported that lack of training in mental health can also lead to “diagnostic overshadowing” (Emma, Liaison Psychiatrist), whereby physical symptoms are misattributed to mental health difficulties in people with SMHDs. For example, one liaison psychiatrist described how delirium, a state of acute confusion common in kidney failure due to the build-up of toxins affecting the brain, can often be overlooked or misunderstood in people with SMHDs:

*The people who present as very flat, they often seem quite depressed, and actually, they're so disengaged and not communicating that no-one has actually asked them, do they know where they are? Or assessed their attention, or orientation. But to us it's still very evident that it's delirium.* – Emma, Liaison psychiatrist

As Liaison Psychiatry have a good understanding of the interface between physical and mental health difficulties, they were described as being able to provide a more nuanced formulation to the renal team, taking biological, psychological and social factors into account. Two liaison psychiatrists described the value of being able to tease apart and make sense of overlapping physical and mental health symptoms:

*For example, some patients with mental disorders may require more analgesia than the team would expect, so I explain to the team why that is and how to support the patients with that. Rather than them either over investigating, “Has something gone wrong here and that's why they have more pain?” Or patient blaming and saying “He doesn’t need any more.”* – Noreen, Liaison Psychiatrist

Participants also described how input from liaison psychiatry is essential for the management of psychotropic medications. Mental health participants stressed how, when considering changing or discontinuing lithium-based medications due to their impact on kidney function, the renal team must take the potential risks for their mental health into account:

*Occasionally there's some patients for whom lithium is the only medication that works and you're making a cost-benefit consideration in terms of transplanting somebody on lithium, and knowing the lithium may have an impact on their new kidney. But yet, in terms of their mental state, the risk-benefit ratio is worth them staying on the lithium* – Noreen, Liaison Psychiatrist

When considering changes in psychotropic medications, participants highlighted the importance of informed consent and taking the preferences of the person with the SMHD into account. Mental health participants also advocated for more awareness in renal teams regarding the potential risks of relapses in mental health, as well as the need for advance planning of support when changing psychotropic medications:

*Relapses can permanently lower a person’s level of functioning and have lasting impacts on their independence. (…) So sometimes it's a bit of a copout from some of the renal teams by not understanding and just giving this advice that we all know. Yes we need to change their meds, but it's HOW do we change it, or how do we manage to change?* – Jack, Community Mental Health Nurse

Some participants suggested providing additional training in SMHDs for healthcare providers, regarding the mind–body link, the interaction between SMHDs and kidney disease, features of specific SMHDs, and psychotropic medications. However, most participants reported it is difficult to incentivise healthcare providers to take part in training because of understaffing and time restraints. Due to the wide range of SMHDs and their presentations, participants recommended having a mental health professional working closely with the renal MDT, who is readily available to healthcare providers and can advise on the care of people with SMHDs on a case by case basis: “I think having a psychologist and psychiatrist in the transplant team is hugely important. Because if it’s really difficult for me to find out what I have to do to help each individual person. Because people have all sorts of difficulties.” (Grace, Consultant Immunologist).

## “When they present to a busy emergency department with a problem, the staff tend to go ‘...psych patient”

Participants described how stigma towards people with SMHDs can lead to discrimination against them in healthcare settings: “I think there is discrimination against people with a mental health diagnosis, and I think it’s quite prevalent.” (Jack, Community Mental Health Nurse). For example, mental healthcare professionals described how the complaints of people with SMHDs are taken less seriously when they present to primary care or emergency services:

*I remember I had one patient who wasn't interacting, so the doctor said "Oh, she's depressed. We need to get her over to the psychiatric unit." And she wasn’t depressed, it turns out she had bacterial encephalitis. And because of lack of intervention an infection was allowed to grow, and she had a horrible death, which was wrong, it should have been picked up earlier. And I know that's an extreme example, but I encounter examples all the time where people with mental health diagnoses, especially those with schizophrenia, are dismissed.* – Jack, Community Mental Health Nurse

The same participants reported that renal healthcare providers are generally very diligent about their patients’ symptoms. However, they expressed concerns that stigma and diagnostic overshadowing may prevent people with SMHDs from being referred to nephrology from other services. Mental healthcare professionals also reported that the negative symptoms of SMHDs, such as lack of motivation, can be negatively interpreted by HPCs: “People with major mood disorder or with psychosis can come across as abrupt or not interested or unmotivated or rude.” (Liam, Liaison Psychiatrist). Similarly, participants reported that although in their experience people with SMHDs did not show more aggression than other patients, there is a perception that people with SMHDs are more dangerous. One haemodialysis nurse reported that this anxiety, combined with uncertainty regarding how to respond to mental health difficulties, can lead to renal healthcare providers’ spending less time with people with SMHDs.

Participants also described how, in an under-resourced system when healthcare providers are burnt out, it can be difficult to accommodate the additional needs of people with SMHDs: “Particularly when they present to a busy emergency department with a problem, the doctors tend to go ‘…psych patient’” (Liam, Liaison psychiatrist). For example, participants described how healthcare providers can be frustrated when people with SMHDs miss appointments, and do not consider the factors that may have contributed to their not attending: “We do not often think about what we could have done to make it easier for them to show up. We just think ‘why would I bother trying to help them again?’” (Liam, Liaison Psychiatrist).

A Community Mental Health Nurse (Jack) reported that education for healthcare providers is an effective way of reducing stigma towards people with SMHDs. He described how he had effectively taught healthcare providers skills in “what to do and what to say,” corrected biases and misconceptions that may negatively impact care for people with SMHDs, and helped the providers feel more comfortable treating people with SMHDs. Mental healthcare providers emphasised the importance of normalizing SMHDs, and treating people with SMHDs with empathy and respect:

*Everyone just goes “oh god, let’s get psychology in” when they hear the person has schizophrenia and is hearing voices. But no, we can all talk to this one person. They’re still human. You can still work with them. It’s still the same skills we use with any other human being* – Isabelle, Clinical Psychologist

## Discussion

For people with kidney disease, it is well documented that those with SMHDs have poorer access to kidney care and worse clinical outcomes (14). To our knowledge, this is the first study to qualitatively assess the barriers and facilitators to kidney care for people with SMHDs. Our findings give insight into a number of ways in which care might be improved for this underserved population. Our results indicate that many people with SMHDs experience additional barriers to kidney care, due to challenges with their mental state, motivation and organisation, cognitive difficulties, or mistrust of the healthcare system. Participants’ accounts describe how the separation of physical and mental healthcare, combined with under-resourcing and understaffing of mental health professionals, make it difficult to support the additional needs of people with SMHDs. Findings also suggests that lack of mental health training for healthcare providers impacts their confidence in their ability to provide care to people with SMHDs. Our results also indicate that system-wide stigma towards people with SMHDs in healthcare contributes to poorer outcomes for this population. Despite these identified barriers, our findings indicate that with adequate social, community and hospital supports, people with SMHDs and kidney disease can have favourable outcomes even in the context of significant impairments and complex presentations. However, effective care for this population requires integrated physical and mental health care, which takes an individualised “whole person” approach to addressing the interaction between kidney disease and mental health (50).

In many ways, participants’ accounts describe how Nephrology services are well suited to manage the needs of people with SMHDs, if they are adequately resourced with mental healthcare professionals. For example, renal healthcare providers typically have long-term relationships with their patients (51), and this continuity of care is associated with better health outcomes for people with SMHDs (52). The close monitoring and proactive follow-up conducted by renal nurses means that fluctuations in mental and physical health are quickly noticed and responded to, and this also reduces the risk of diagnostic overshadowing. Furthermore, in contrast to the stigmatising and pessimistic attitudes displayed by healthcare providers elsewhere (6, 53), most renal healthcare participants in this study held positive views about people with SMHDs and their treatment outcomes.

Barriers to kidney care for people with SMHDs were identified at the person, provider and systems, level, and were largely consistent with those described for people with SMHDs accessing other forms of healthcare (5, 54). Several barriers specific to kidney care for people with SMHDs were identified for the first time, including the high burden of adherence to recommendations regarding diet, fluid intake, medications, and dialysis treatment. Research has shown that the most effective ways to improve medication adherence for people with SMHDs are tailored to the person’s specific needs, and take into account their motivational, cognitive and functional difficulties (55). They also use a problem-solving approach to identify barriers to adherence, and address ambivalence that people with SMHDs have towards committing to medication regimens (55). Interestingly, our results indicate that the most vulnerable cohort of people with SMHDs with kidney failure are not those with the most significant impairments, but those who are not deemed “unwell enough” to be in fully supported accommodation and have few social supports. As evidenced in other chronic conditions such as diabetes, this highlights the importance of family and other external supports in promoting adherence for people with SMHDs (56). Tailored environmental support systems combined with cognitive adaptation training have been shown to significantly improve medication adherence in people with schizophrenia (57). However, to our knowledge there are no studies addressing adherence in people with SMHDs and kidney failure. Given the burden of adherence and its critical impact on outcomes, research addressing adherence for people with SMHDs and kidney failure who are living in the community should be prioritized.

The risks associated with non-adherence to treatment, combined with high rates of suicide and self-harm in people with SMHDs (58), make people with SMHDs and kidney failure an extremely vulnerable group. The link between depression and poorer adherence in people with kidney failure is well documented (59). Results highlight the need for close monitoring of mental state for people with SMHDs, and access to on-site mental health professionals who can facilitate appropriate interventions in a timely manner. Individualised care plans should be available to all clinicians working with individuals with fluctuating mental states. In addition to having crisis and contingency plans, individualised care plans should clearly outline agreed intervention strategies for mental and physical health, strategies for self-management, and advance directives or statements the person has made (60). Care plans should be developed collaboratively by members of the nephrology MDT, mental health team, the person with the SMHD, and their family or carers as appropriate.

Renal healthcare participants lacked confidence in their understanding of mental health and their ability to care for people with SMHDs, consistent with previous research (33). Given the wide range of SMHDs and the complexity of kidney care for this cohort, findings indicate that renal healthcare providers require individualised input from mental healthcare professionals on a case-by-case basis. Our results add to existing evidence that close collaboration with mental health professionals increases physical healthcare providers’ competence and proactive engagement when working with people with SMHDs (61). Findings indicate that Nephrology departments should have comprehensive and proactive multidisciplinary team-based care including psychiatry, psychology, social work, and clinical nurse specialists, similar to the approach outlined in the Irish National Cancer Strategy 2017–2026 (62). At minimum, timely and frequent communication between renal and mental healthcare providers is necessary to ensure safe and effective treatment for people with SMHDs.

Another issue specific to people with SMHDs and kidney disease is the management of lithium-based psychotropic medications. Lithium is considered by many to be the “gold standard” treatment for bipolar disorder, and appears to be superior to other mood stabilisers in preventing suicide in people with SMHDs (63, 64). Long-term lithium use has been associated with higher rates of chronic kidney disease, although there is little evidence that discontinuing lithium decreases risk of kidney failure (65, 66). In line with international best practice guidelines (67), mental health participants in this study reported they regularly monitor the kidney function of clients on lithium. However, our results indicate that some mental healthcare providers need additional support from specialist Nephrology services to inform preventative measures for people with SMHDs in the early stages of kidney damage. This study also adds to existing evidence that the decision to change or discontinue lithium treatment for people with SMHDs and kidney disease requires careful consideration of the potential risks and benefits for each person (65, 68). Renal healthcare providers should be aware of the increased risk of mania, depression and suicide following removal of lithium (69), and consider how these risks can be managed before discontinuation. Because of the uncertainties involved, the decision making process must include the people with SMHDs, their family or carers, nephrologists and mental health professionals.

Findings highlight the impact of stigma towards people with SMHDs in healthcare, and how it can lead to dismissal of symptoms, diagnostic overshadowing, and less effective care (70). Stigma occurs on multiple levels throughout the healthcare system, including intraindividual (e.g., patient self-stigma and reluctance to seek care), interpersonal (e.g., negative attitudes and discriminatory behaviours by clinicians) and structural (e.g., investment of resources) (6). System-wide interventions to reduce healthcare providers’ stigma may improve people with SMHDs’ access to Nephrology specialists and the quality of kidney care they receive. Our findings add to evidence that interventions should teach skills in “what to do” and “what to say” when interacting with people with SMHDs, and target clinicians’ unconscious biases and false beliefs that may be having a negative impact on care (71). Research suggests that most effective stigma-reducing programs also use social contact, whereby healthcare providers hear testimonies from people with lived experience of SMHDs (72).

### Strengths, limitations and areas for future research

To our knowledge, this is the first study investigating barriers and facilitators to effective kidney care for people with SMHDs. The perspectives of a range of healthcare and mental healthcare professionals with differing roles and perspectives add valuable insights into how kidney care for people with SMHDs might be improved. However, as the perspectives of people with SMHDs and their carers/family members may be very different from those of professionals, further research involving their perspectives is crucial to understanding the full range of barriers and facilitators to care for this population. Furthermore, as this was a relatively small sample of healthcare providers based in Ireland, further research is required to determine whether findings are transferrable to kidney care in other settings. The current study also did not include the perspectives of General Practitioners, who may have additional insights into the barriers and facilitators to effective kidney care for this population.

This study focused on kidney care for a wide range of mental health-related diagnoses and presentations. Research focusing on kidney care for people with specific difficulties (e.g., psychosis, mood fluctuations, strongly held beliefs, etc.) may give more nuanced and specific insights into how to improve clinical outcomes for these individuals. Given the concern about risk among professionals in the current study, assessing the rates of suicide and loss of contact with healthcare services for people with SMHDs and co-occurring kidney disease should be a research priority. Lastly, further research is needed to determine how to effectively support adherence in people with SMHDs and kidney failure living in the community, in the context of limited social supports.

## Conclusion

As evidenced in other medical conditions, many people with SMHDs require additional support to access kidney care, due to fluctuating mental state, problems with motivation and organisation, cognitive difficulties, or anxiety and mistrust of the healthcare system. Our results highlight the need to understand the specific limitations and challenges of each person with a SMHD, so that individualised supports can be provided. Findings indicate that, with adequate social, community and hospital supports, people with SMHDs and kidney disease can have favourable outcomes even in the context of significant impairments and complex presentations. However, the separation of physical and mental healthcare, combined with under-resourcing and understaffing, often make it difficult for renal settings to meet the full range of needs of people with SMHDs with kidney disease. Lack of ongoing education in mental health for renal healthcare providers and the resulting discomfort treating people with SMHDs can also negatively impact outcomes for this population. This study gives insight into a number of ways in which care can be improved for people with SMHDs, including integrated physical and mental health care, which takes a “whole person” approach to addressing the interaction between kidney disease and mental health. Our results indicate that renal departments should have multidisciplinary team-based care, including psychiatry, psychology, social work, and clinical nurse specialists. Communication and coordination between renal and mental healthcare providers is also necessary to ensure safe and effective kidney care for people with SMHDs. As this study focused solely on the perspective of healthcare providers, further research including the perspectives of people with SMHDs with kidney disease and their family members is needed to inform the provision of care for this population.

## Data availability statement

The datasets presented in this article are not readily available because the de-identified transcripts include potentially identifying information. Requests to access the datasets should be directed to clodagh.cogley@ucdconnect.ie.

## Ethics statement

The studies involving human participants were reviewed and approved by Human Research Ethics Committee – Humanities. University College Dublin, Ireland HS-21-19-Cogley-Dalton. The patients/participants provided their written informed consent to participate in this study.

## Author contributions

CCo, PD’A, JB, and KB conceived and planned the study. AS, JH, and PC, facilitated recruitment. CCo collected the data. CCo and CCa conducted analyses, with input from PD’A, JB, and KB. CCo wrote the manuscript with input from all authors. All authors contributed to the article and approved the submitted version.

## Funding

This research was funded by the Irish Research Council (GOIPG/2021/474) (CCo) and the Central Remedial Clinic (CCo).

## Conflict of interest

The authors declare that the research was conducted in the absence of any commercial or financial relationships that could be construed as a potential conflict of interest.

## Publisher’s note

All claims expressed in this article are solely those of the authors and do not necessarily represent those of their affiliated organizations, or those of the publisher, the editors and the reviewers. Any product that may be evaluated in this article, or claim that may be made by its manufacturer, is not guaranteed or endorsed by the publisher.
