# Supplementary material for: Improving kidney care for people with severe mental health difficulties: a thematic analysis of twenty-two healthcare providers’ perspectives
Source: Front Public Health. 2023 Jun 28;11:1225102. doi: 10.3389/fpubh.2023.1225102 (PMC10338099; doi:10.3389/fpubh.2023.1225102)
Supplement: Supplementary file 1 [file Data_Sheet_1.docx]

**Semi-structured interview schedule:**

Do you have experience providing care to people with kidney disease and SMHDs (e.g. schizophrenia, bipolar disorder, psychosis, major depression)?

Roughly how many ESKD patients with SMHD have you provided healthcare to? What kinds of mental health difficulties have they had?

**Access to care**

In your opinion, do individuals with SMHDs have difficulty accessing quality healthcare for their kidney disease?

Does having a SMHD make any aspect of kidney care more difficult? Why?

**ESKD Diagnosis**
At the diagnosis stage, what are the biggest barriers to effective healthcare for people with SMHDs?

At the diagnosis stage, what helps care go well for people with SMHDs?

Is there any way care could be improved for ESKD patients with concurrent SMHDs at this stage?

**Dialysis:**

In your experience, do patients’ SMHDs impact dialysis?

In your experience, what is the most difficult aspect of dialysis for these patients?

What are the biggest barriers to care at this stage?

What helps dialysis go well for these patients?

Do patients with SMHDs find adherence more challenging?

**Transplant**:

What are the barriers to transplant for people with SMHDs?

Are you aware of any patients with SMHDs who have received a kidney transplant?

Are you aware of any patients with SMHDs who have been refused a kidney transplant? Do you know why?

What helps care go well for people with SMHDs at this stage?

Is there any way this process could be made easier for individuals with SMHDs?

**Treatment of SMHD (if not covered)**

How do you typically become aware of an individual’s SMHD?

When you learn of a patient’s SMHD, does this impact the treatment offered to them in any way?

Do the healthcare team ever discuss how the mental health difficulty might affect their kidney treatment? Or vice versa?

Are there any additional supports in place for these patients?

**HCPs’ knowledge of mental health difficulties (if not covered)**

How are psychiatric symptoms typically managed?

How comfortable, overall, do you feel treating patients with SMHD and ESKD?

**Mental health care services (if not covered)**

Are ESKD patients with SMHDs typically involved with mental health care services?

Is there typically any contact between mental health services and the renal team?

Do you have experience working with psychiatry, psychology or social work, in order to provide care to individuals with ESKD and SMHD?

**Closing questions**

What do you think patients with SMHDs find most difficult about kidney care? How could this be improved?

What do you think healthcare providers find most difficult when working with patients with SMHDs? How could this be improved?

**Please share any ideas of how you think that we could improve the care of patients with SMHD and ESKD.**

In closing, is there anything else that you’d like to add that is relevant to the care of ESKD patients with mental health difficulties? Anything that we didn’t ask?
